# Characterization of the CqCAMTA gene family reveals the role of *CqCAMTA03* in drought tolerance

**DOI:** 10.1186/s12870-022-03817-0

**Published:** 2022-09-07

**Authors:** Xiaolin Zhu, Baoqiang Wang, Xiaohong Wei, Xuefeng Du

**Affiliations:** 1 College of Agronomy, Gansu Agricultural University, Lanzhou, 730070 China; 2 Gansu Provincial Key Laboratory of Aridland Crop Science, Gansu Agricultural University, Lanzhou, 730070 China; 3 Present Address: College of Life Science and Technology, Gansu Agricultural University, Lanzhou, 730070 China

**Keywords:** Quinoa, CAMTA gene family, Subcellular localization, Yeast self-activation, Transgenic *Arabidopsis*, drought stress

## Abstract

**Background:**

Calmodulin-binding transcription activators (CAMTAs) are relatively conserved calmodulin-binding transcription factors widely found in eukaryotes and play important roles in plant growth and stress response. CAMTA transcription factors have been identified in several plant species, but the family members and functions have not yet been identified and analyzed in quinoa.

**Results:**

In this study, we identified seven CAMTA genes across the whole quinoa genome and analyzed the expression patterns of CqCAMTAs in root and leaf tissues. Gene structure, protein domain, and phylogenetic analyses showed that the quinoa CAMTAs were structurally similar and clustered into the same three major groups as other plant CAMTAs. A large number of stress response-related cis-elements existed in the 2 kb promoter region upstream of the transcription start site of the CqCAMTA genes. qRT-PCR indicated that CqCAMTA genes were expressed differentially under PEG treatments in leaves, and responded to drought stress in leaves and roots. In particular, the *CqCAMTA03* gene strongly responded to drought. The transient expression of CqCAMTA03-GFP fusion protein in the tobacco leaf showed that *CqCAMTA03* was localized in the nucleus. In addition, transgenic *Arabidopsis* lines exhibited higher concentration levels of the antioxidant enzymes measured, including POD, SOD, and CAT, under drought conditions with very low levels of H_2_O_2_ and MDA. Moreover, relative water content and the degree of stomatal opening showed that the transgenic *Arabidopsis* lines were more tolerant of both stress factors as compared to their wild types.

**Conclusion:**

In this study, the structures and functions of the CAMTA family in quinoa were systematically explored. Many CAMTAs may play vital roles in the regulation of organ development, growth, and responses to drought stress. The results of the present study serve as a basis for future functional studies on the quinoa CAMTA family.

**Supplementary Information:**

The online version contains supplementary material available at 10.1186/s12870-022-03817-0.

## Introduction

In the process of plant growth and development, plants respond to various environmental changes, and transcription factors play a crucial regulatory role. They participate in regulating the transcriptional activity of target genes by recognizing and binding the corresponding cis-acting elements of target genes [1]. The calcium (Ca^2+^) signal, as a core sensor and regulator, takes part in diverse physiological processes in plants, including various responses to biological and non-biological stimuli, and is of great significance to the regulation of gene transcription [2–4]. In the process of Ca^2+^ signal transduction, the decoding of stimulus response coupling involves a set of Ca^2+^ sensor proteins or Ca^2+^ binding proteins [3]; these proteins usually have a helix-loop-helix structure [5]. There are three main types of Ca^2+^ sensor proteins in plants: calmodulin (CaM) proteins, calcium-dependent protein kinases (CDPKs), and calcineurin B-like proteins [6, 7]. CaM is considered to be a typical Ca^2+^ binding protein. The CaM binding domain can directly bind to DNA and activate transcription, or interact with other transcription factors without DNA binding, and so act as a co-activator of transcription [8]. CaMs in plants can regulate more than 90 transcription factors [9], such as CAMTAs, MYBs, WRKY IIDs, bZIPs, and NACs [10].

CAMTAs are a family of transcriptional activators that can interact with CaM in plants [11, 12]. First isolated in tobacco and named *NtER1* (ethylene response 1) because of its rapid increase in expression under the induction of ethylene, *NtER1* is developmentally regulated and causes senescence and death, but NtER1 is in essence a CAMTA protein [11]. Because this family of genes can have rapidly induced expression as a result of environmental signals (extreme temperature and salt), hormones (C2H4 and ABA), and signal molecules (MeJA, H_2_O_2_, and SA), AtCAMTA was also named AtSR (*Arabidopsis thaliana* signal-responsive genes) [13]. CAMTA in rice is called OsCBT (*Oryza sativa* CaM-binging transcription factor) [14]. All members of this family have a CaM binding domain. From the N-terminal to C-terminal, they contain a NLS (nuclear localization signal) CG-1 DNA binding domain, immunoglobulin-like DNA binding domain (TIG), ankyrin repeating sequence (ANK), a varying number of IQ motifs (IQXXXRGXXXR), and a Ca^2+^-dependent calmodulin binding domain (CaMBD) [13, 15]. The CG-1 domain, composed of approximately 130 amino acids, is unique to eukaryotic multicellular organisms, and the protein encoded by it can bind to DNA sequences containing CGCG [13]. TIG domains exist in many transcription factors with different functions, can bind non-specifically with DNA, and are also involved in protein dimerization [16]. The ANK repeat sequence is a repetitive tandem module of many eukaryotic cell proteins and viruses, and is also involved in the interaction between proteins [16]. CaMBD can bind to the Ca^2+^/CaM complex [14]. The IQ motif has a repeating motif IQXXXRGXXX, and its binding to calmodulin can be independent of Ca^2+^ [14].

At present, CAMTA families have been identified in many species, such as *Arabidopsis thaliana* [17], *Oryza sativa* [14], *Solanum lycopersicum* [18], *Zea mays* [19], *Linum usitatissimum* [20], and *Musa nana* [21]. Among these, *AtCAMTA1* and *AtCAMTA5* can regulate the expression of *AVP1* in pollen. *AVP1* can regulate plant growth and development by regulating the transport of auxin [22]. *NtER1* (CAMTA) is involved in the aging process caused by ethylene [11]. In *Arabidopsis*, *CAMTA1*, *CAMTA2*, and *CAMTA3* act synergistically to inhibit the expression of genes participating in salicylic acid (SA) biosynthesis and mediated immunity, thereby improving plant freezing tolerance [23]. *AtCAMTA1* regulates drought recovery by regulating the expression of TFs (DREB, bHLH, and MYB) and the response of ABA [24]. Galon [11] found that *AtCAMTA1* participates in auxin signal transduction and responds to abiotic stress. Li [25] found that *SlSR1L* and *SlSR2L* in tomato CAMTA/SR can positively regulate drought resistance. Overexpression of *GmCAMTA12* in soybean improved the drought resistance of hairy roots [26]. Studies have shown that *CAMTA3/SR1* and *CAMTA6* can directly inhibit the expression of salt-related genes to negatively regulate the salt tolerance of plant [27, 28].

Quinoa (*Chenopodium quinoa* Willd) is an annual dicot in the family Amaranthaceae. It is a heterotetraploid (2n = 4X = 36). Quinoa is native to the Andes of South America and has the tolerance for cold, salt, and drought. Quinoa has rich and comprehensive nutritional value, and is known as the “mother of food”. It is the only single plant that can nearly meet the basic nutritional needs of the human body [29]. Analysis of the whole genome sequence of high-quality quinoa in 2017 opened a new window for the research of quinoa germplasm resources and its molecular design and breeding. Research on quinoa transcription factors have included GRAS, NAC, and LIM, but there are no reports yet on quinoa CAMTA transcription activators. Therefore, in order to understand the diversity of the quinoa CAMTA transcriptional activator family, this study is based on the quinoa genome database, using bioinformatics methods to analyze all members of the quinoa CAMTA gene family, and systematically analyze the protein physicochemical properties of the CAMTA gene family, gene structure, phylogenetic analysis, and promoter sequence analysis, and analysis of quinoa leaf expression under different stress treatments. This lays a foundation for in-depth understanding of the quinoa CAMTA transcriptional activator family and exploring the response mechanism of plants under stress conditions, and provide genetic resources for quinoa genetic breeding.

## Materials and methods

### Identification of quinoa CAMTA gene family

Quinoa genome data, CDS sequence, GFF annotation information, and amino acid sequence files are from PhytozomeV12 database (https://phytozome.jgi.doe.gov/pz/portal.html). The *Arabidopsis* CAMTA protein sequence is from Ensembl Plants (http://plants.ensembl.org/index.html) [30], to predict the quinoa CAMTA (CAMTA domains: CG-1, TIG domain, Ankyrin repeats, IQ). The Hidden Markov Model (HMM) profile of the CG-1 domain (PF03859), the ANK repeat domain (PF00023), TIG (PF01833) and the IQ domain (PF00612) sequences were downloaded from the PFAM database [31]. We used the HMMER search tool with E value <= 0.0001 to check the protein sequence of quinoa [32]. We submitted the CAMTA protein sequence obtained from the preliminary screening to NCBI-CDD (Conserved Domains Database) (https://www.ncbi.nlm.nih.gov/cdd/) and InterProScan (http://www.ebi.ac.uk/interpro/search/sequence-search) database to further determine the conserved domain. Finally, the genes with the complete domains of CG-1, ANK repeat, TIG and IQ were regarded as putative CAMTA genes. The basic physical and chemical properties of CqCAMTA proteins were analyzed by ExPASy with the default parameters (https://web.expasy.org/compute_pi/), and the subcellular location prediction of this family protein was carried out by Plant-mPLoc with the default parameters (http://www.csbio.sjtu.edu.cn/bioinf/plant-multi/) [33].

### Construction of phylogenetic tree, analysis of its structure domain and chromosome location analysis

The CAMTA protein sequences of *Arabidopsis thaliana*, grape, spinach, and pepper are used to construct phylogenetic trees, they are from the Ensembl Plants database (http://plants.ensembl.org/index.html). First, the ClustalW program in MEGAX was used to compare the amino acid sequences, and the parameters are defaulted. Second, in MEGAX, the neighboring method was used to construct a phylogenetic tree, the bootstrap method was used with repetitions set to 1000, model was p-distance, and missing date treatment was pairwise deletion, number of threads was four, rates among sites was uniform rates [34]. Finally, the illustration of the evolutionary tree was through Evolview (http://120.202.110.254:8280/evolview) [35]. In addition, the visualization of the conserved domains of CqCAMTA protein were performed by TBtools software. In addition, through the GFF annotation file, TBtools software was used to analyze the chromosome location of the identified CqCAMTA genes.

### Analysis of CqCAMTA gene structure and conserved motif

Quinoa genome data, CDS sequence, GFF annotation information, and protein sequence files are from PhytozomeV12 database (https://phytozome.jgi.doe.gov/pz/portal.html), using the quinoa GFF annotation file (Table S1), the CqCAMTA genes structure was analyzed through the website Gene Structure Display Server 2.0 (GSDS 2.0, http://gsds.cbi.pku.edu.cn/) [36]. The basic motif of CqCAMTA protein (Table S1) was analyzed on the Multiple Em for Motif Elicitation (MEME) [37] website. The parameters were as follows: 5–100 optimum width of amino acids and and the E value was less than 1e^− 20^, 10 maximum number of motifs. The picture was displayed by using TBtools.

### Analysis of Cis-elements and construction of protein interaction network diagram

In order to further study the sequence of the promoter region of the CqCAMTA genes, the 2 kb sequence upstream of the transcription start site of the CqCAMTA genes was obtained from the PhytozomeV12 database (https://phytozome.jgi.doe.gov/pz/portal.html), and the sequence of 2 kb upstream of the transcription start site of the CqCAMTA genes was obtained through PlantCARE (http://bioinformatics.psb.ugent.be/webtools/plantcare/html/) [38] and analyzed for the types of cis-elements components they contained, and these results were visualized by Excel 2003. In addition, based on the *Arabidopsis* CAMTA protein, the well-characterized model plant was the subject organism (combined score ≥ 0.4). the interaction network diagram of CqCAMTA was constructed through STRING (https://www.string-db.org/) [27].

### Plant material and treatments

L-1 (Longli NO.1 from Gansu Academy of Agricultural Sciences) was used as the test material. The experiment was carried out in the Crop Genetics and Breeding Laboratory of Gansu Agricultural University from June to October 2021. On June 10, 2021, we selected plump quinoa seeds of the same size, disinfect the surface with 5% NaClO for 20 minutes, rinsed with water 5 times, dried them, and sowed them in a plastic pot with a diameter of 20 cm and a height of 14 cm. As the substrate, 20 seeds were sown in each pot, and then placed in a solar greenhouse for cultivation after permeable irrigation. The light intensity in the greenhouse was 400-600 μmol·m-2·s-1, day/night temperature (24 ~ 37) °C/(16 ~ 22) °C, humidity (70% ± 10%). When the seedlings grew to 1 leaf and 1 heart, seedlings were thinned to 10 in each pot and irrigated with 300 mL 1/2 Hoagland nutrient solution to supplement nutrients. Stress treatment was performed when the seedlings were at the 4-leaf stage, with three replicates for each treatment. PEG treatment: watering the seedlings from the roots with 20% PEG6000, 300 mL per pot. Drought treatment: no watering from the beginning of the test. Quinoa seedling leaves were taken 0, 3, 6, 9, 12, 24, and 48 h after treatment with PEG. 0, 3, 5 and 7 d after drought treatment, quinoa seedling leaves and roots were collected and stored at − 80 °C for subsequent experiments.

### RNA extraction, cDNA synthesis and real-time fluorescence quantitative PCR

RNA of leaves and roots was extracted using NucleoSpin® RNA Kit (Macherey–Nagel, Germany). NanoDrop Lite UV-vis spectrophotometer was used to detect the quality of RNA. The synthesis of cDNA used the Superscript TM III reverse transcriptase kit (Perfect Real Time, Takara Biomedical Technology Co., Ltd. (Beijing, China)). RT-qPCR analysis was performed with the ABI-VIIA 7 real-time PCR system (American Applied Biosystems) using 2 quantitect-sybr-green-pcr-mix (Qiagen). The primer sequences of the CqCAMTA genes and housekeeping gene are shown in Table S2. Program: After pre-denaturation for about 10 min at 95 °C, 40 cycles of 15 s at 95 °C, 20 s at 60 °C and 20 s at 72 °C were applied. The relative expression level was measured by the 2^-△△Ct^ method [39].

### Subcellular localization and transcriptional activation assay of *CqCAMTA03*

The coding sequence (CDS) of *CqCAMTA03* was amplified using the special primers. Amplified *CqCAMTA03* DNA were digested with 5′ NcoI and 3′ SpeI restriction enzymes, respectively, and then inserted into the NcoI and SpeI-digested pCAMBIA1302-35S-EGFP vectors, respectively, to produce pCAMBIA1302-35S-FXRE::GFP and pCAMBIA1300-35S-NYUW::GFP. Both recombinant plasmids were transformed into *Agrobacterium tumefaciens* strain GV3101. The *Agrobacterium* of the prepared recombinant expression vector pCAMBIA1302-35S-EGFP was used as the infection solution, and the *Agrobacterium* solution of the pCAMBIA1302-35S-EGFP empty vector was used as the control (CK) to carry out the infection and transformation of *Nicotiana tabacum*. GFP fluorescence was observed using an Airyscan confocal laser scanning microscope (ZEISS LSM 880, Carl Zeiss, Jena, Germany).

*CqCAMTA03* was inserted into the pGBKT7 vector using NdeI and BamHI to generate bait construct pGBKT7-CqCAMTA03. pGBKT7-Lam and pGBKT7–53 served as the negative and positive controls, respectively. Yeast strain AH109 was transformed with the three plasmids and grown in a selection medium lacking tryptophan (SD/−Trp). The positive clones were then obtained and cultured in SD/−Trp/−Ade/−His/+X-α -Gal medium.

### Generation of transgenic *Arabidopsis* plants overexpressing *CqCAMTA03*

The coding sequence of *CqCAMTA03* (no stop codon) was inserted into the pCAMBIA1302-35S-EGFP vector. Then, the gateway reaction was applied to pCAMBIA1302 containing CaMV 35S promoter fused with GFP to generate pCAMBIA1302-CqCAMTA03. The *Agrobacterium*-mediated transformation method was performed to generate the transgenic *Arabidopsis* lines [40]. Positive seedlings were selected by spraying Basta (1:1000). Homozygous seedlings were established from the T3 generation and used for the subsequent analyses.

### Phenotype observations and physiological index measurements in transgenic *Arabidopsis*

The overexpressing and wild type *Arabidopsis* seedlings were evaluated under drought conditions. *Arabidopsis* plants were placed in a growth chamber and grown under normal conditions (16 h/8 h light/dark, 25/18 °C day/night). For the drought tolerance assay, 4-week-old plants of the WT and transgenic lines grown in the soil mixture containing vermiculite and Metro-Mix were cultured without watering. After 10 d of drought treatment, the rosette leaves were collected for RNA extraction and determination of physiological parameters. All the experiments mentioned above were replicated five times. MDA [41], H_2_O_2_ [42] and proline content [43], and the activity of SOD [44], CAT [45], and POD [46] were measured. The relative water content of leaves was measured as the method described by Barrs [47].

### Statistical analysis

The experiments were done in three biological replicates, and the data were statistically analyzed by the analysis of variance (ANOVA) procedure [48], using Origin. The least significant difference test (*P* ≤ 0.05) was used for mean comparison.

## Results

### Identification of quinoa CAMTA gene family

In this study, seven CAMTA members were identified in quinoa, and named *CqCAMTA01*-*CqCAMTA07*. The basic physical and chemical properties of the members of the CqCAMTA family were analyzed (Table 1), and the results showed that the amino acid length was between 931 aa (CqCAMTA03) and 1000 aa (CqCAMTA07). The relative molecular weight of the proteins was between 105,392.45 Da (CqCAMTA03) and 111,607.20 Da (CqCAMTA07). The theoretical isoelectric point ranges from 5.40 (CqCAMTA06) to 7.15 (CqCAMTA01). The theoretical isoelectric point of CqCAMTA01 is greater than 7, which makes it a basic protein. The remaining six CqCAMTA proteins (CqCAMTA02-CqCAMTA07) are acidic proteins. The instability coefficient was between 39.31–46.18, CqCAMTA02 is a stable protein (protein with an instability coefficient less than 40). The aliphatic index is 74.20-79.34. The hydrophobicity values of the seven CAMTA proteins < 0, illustrating that the seven CqCAMTA genes all encode hydrophilic proteins. The prediction of subcellular localization showed that seven CAMTA proteins were localized on the nucleus.

**Table 1:** Physical and chemical characteristics of the seven CqCAMTAs

Gene accession No	Gene	Size (aa)	Molecular weight (D)	Isoelectric point	Instability index	Aliphatic index	GRAVY	Subcellular Localization
AUR62016135-RA	*CqCAMTA01*	932	105,523.46	7.15	40.58	78.51	−0.445	nucleus
AUR62002002-RA	*CqCAMTA02*	990	111,890.96	6.47	39.31	75.46	−0.582	nucleus
AUR62041410-RA	*CqCAMTA03*	931	105,392.45	6.97	40.24	78.07	−0.424	nucleus
AUR62003800-RA	*CqCAMTA04*	986	111,419.44	6.61	43.90	74.20	−0.595	nucleus
AUR62013128-RA	*CqCAMTA05*	964	107,247.20	5.46	45.82	75.47	−0.511	nucleus
AUR62005638-RA	*CqCAMTA06*	970	108,219.78	5.40	42.43	79.34	−0.527	nucleus
AUR62010136-RA	CqCAMTA07	1000	111,607.20	5.76	46.18	76.07	−0.525	nucleus

GRAVY represent Grand average of hydropathicity

### Phylogenetic tree construction, conserved domain analysis, and chromosome location analysis

**Fig. 1:**
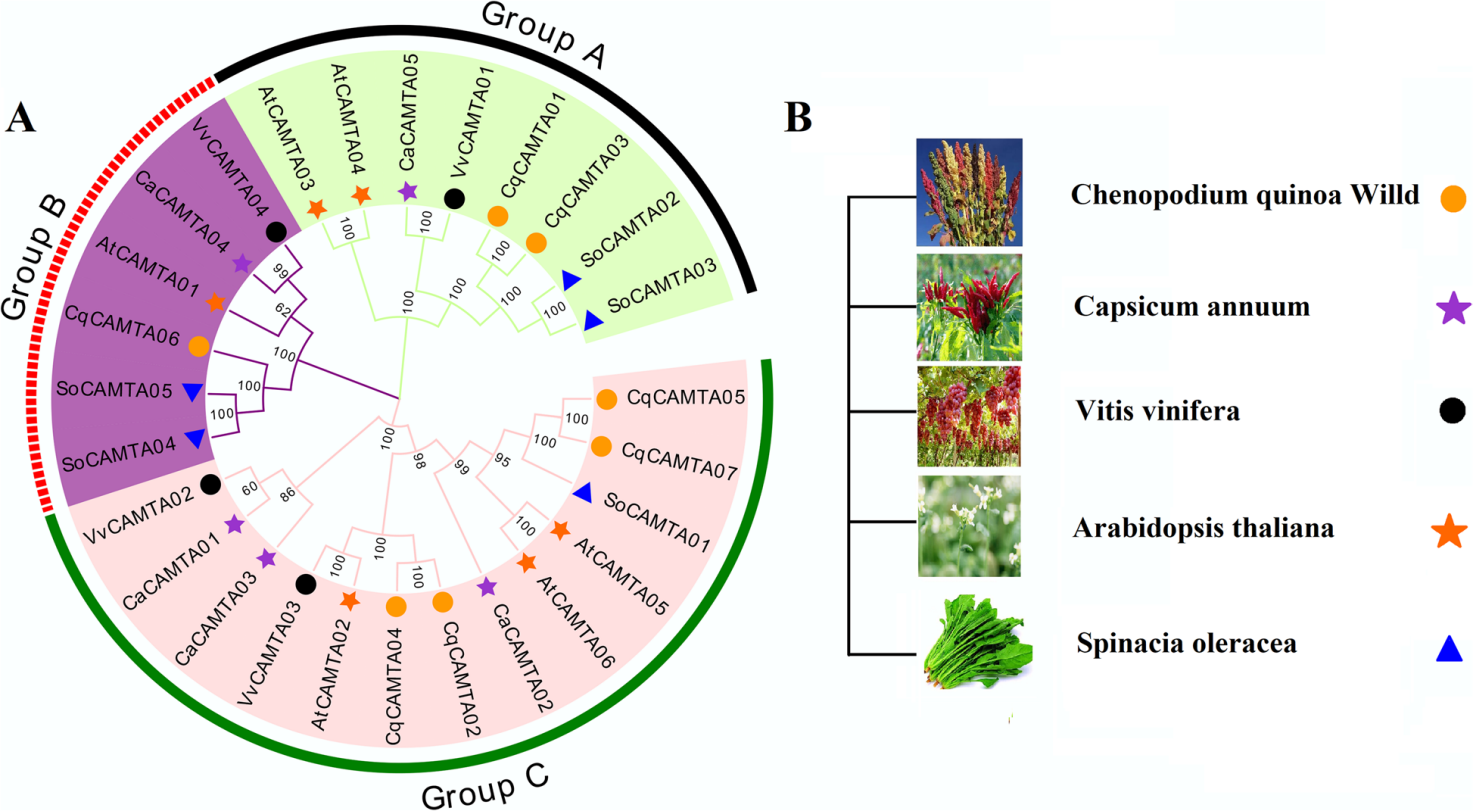
Phylogenetic analysis and grouping of the CAMTA protein family. **A** The phylogeny tree of four VvCAMTAs, five CaCAMTAs, five SoCAMTAs, six AtCAMTAs and seven CqCAMTAs. It was constructed by the neighbor-joining (NJ) method, and the cutoff value of the condensed tree was 60%. three subfamily were highlighted with a specific background color. **B** Identification of the five species included in the phylogenetic tree

For a closer study of evolutionary relationships between the quinoa CAMTA genes and those of other species, a phylogenetic tree of members of the CAMTA family from quinoa, *Arabidopsis*, pepper, spinach, and grape was constructed. The results show (Fig. 1A-B, Table S3) that 27 CAMTA families clustered in three sub-families (Group A-Group C). Seven CqCAMTA genes exist in three subfamilies: the Group A subfamily contains eight CAMTA genes (two CqCAMTA: *CqCAMTA01* and *CqCAMTA03*), the Group B subfamily contains six CAMTA genes (CqCAMTA: *CqCAMTA06*), and the Group C subfamily contains four CqCAMTA genes (*CqCAMTA02*, *CqCAMTA04*, *CqCAMTA05,* and *CqCAMTA07*). There are three pairs of paralogous genes in quinoa (*CqCAMTA01*/*CqCAMTA03*, *CqCAMTA02*/*CqCAMTA04*, *CqCAMTA05*/*CqCAMTA07*). From the phylogenetic tree, it is clear that the CqCAMTAs are closely related to those in spinach. In addition, we found that all 7 CAMTA proteins had conserved domains of CG-1, TIG, ANK and IQ (Fig. S1). According to the GFF annotation file of the quinoa genome, the seven CqCAMTA genes identified were mapped to the chromosomes (Fig. S2). The results showed that the seven CqCAMTA genes were located on six different chromosomes, and chromosome 7 contained two CqCAMTA genes (*CqCAMTA01* and *CqCAMTA02*), while each of the remaining five chromosomes contained one gene.

### CqCAMTA gene structure and conservative motif analysis

**Fig. 2:**
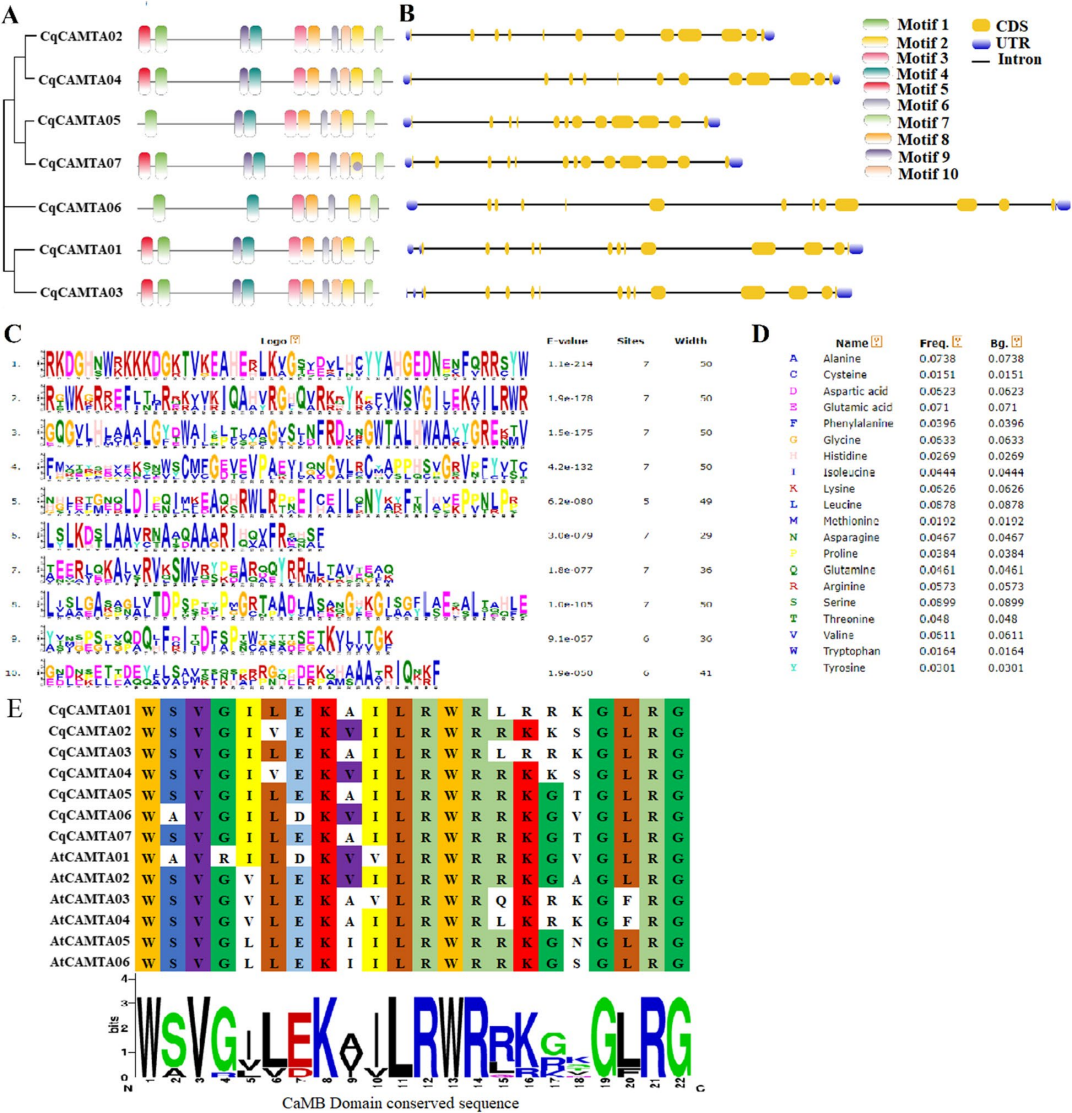
Classification, Conserved motifs and exon–intron distribution of seven CqCAMTAs. **A** The phylogeny tree of seven CqCAMTAs and conserved motifs in seven CqCAMTAs. **B** The exon-intron distribution of seven CqCAMTAs. The exons, introns, and untranslated regions (UTRs) were indicated with yellow boxes, black lines and blue boxes, respectively. The length of the introns and exons can be estimated based on the bottom scale. **C** Alignment of repeated sequences in each motif. The 10 different motifs are highlighted in different colored boxes numbered 1 to 10. **D** The proportion of amino acids. **E** Alignment showing the conserved motif sequence of the CqCAMTA TFs. Each conserved residue at the definite position along the row (throughout the orthologues) is shaded in unique color

To better understand the diversity of CqCAMTA proteins, the basic motifs of CqCAMTA proteins were predicted by MEME, and the results (Fig. 2A, C-E) showed that five CqCAMTA proteins (CqCAMTA01, CqCAMTA02, CqCAMTA03, CqCAMTA04, and CqCAMTA07) contained ten conserved motifs (Motif 1-Motif 10). These were not retrieved in CqCAMTA05 and CqCAMTA06. In addition, it can be observed that the conserved motifs of closely related proteins are essentially the same. In addition, the gene structure of the members of the CqCAMTA family was analyzed (Fig. 2B), and the seven CqCAMTA genes had non-coding sequences at the 5′ and 3′ ends, all genes had an intron-exon genetic structure, and the number of exons was between 12 and 13, indicating that the gene structure is highly conserved. Three CqCAMTA genes (*CqCAMTA02*, *CqCAMTA04,* and *CqCAMTA05*) have 12 exons that are highly conserved; four CqCAMTA genes (*CqCAMTA01*, *CqCAMTA03*, *CqCAMTA06,* and *CqCAMTA07*) have 13 exons. Genes in the same subfamily have similar gene structures, such as *CqCAMTA05* and *CqCAMTA07*. According to the prediction of motifs and gene structure analysis, although the number of conserved motifs and the length of exons and introns have certain differences, the conserved motifs and gene structures of members of the same subfamily are highly conserved.

### Analysis of Cis-acting elements and construction of protein interaction network diagram

**Fig. 3:**
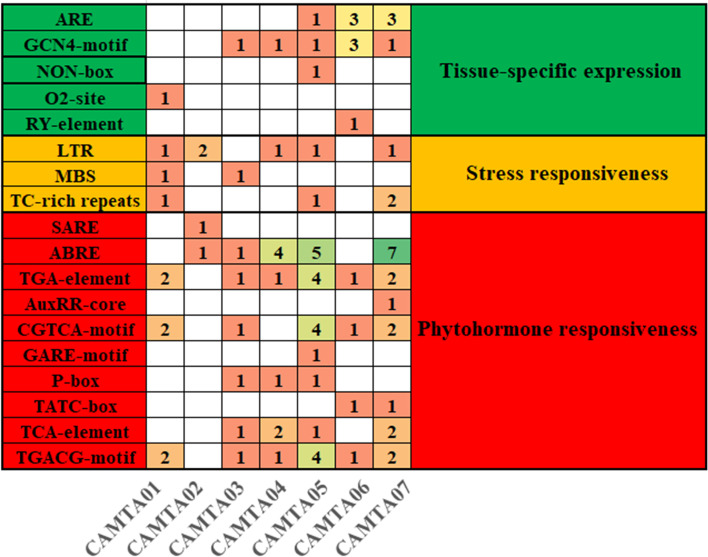
Cis-acting elements in seven CqCAMTAs promoters. The number (one-seven) indicated the number of genes corresponding to the specific cis-acting elements

Cis-acting elements are a type of nucleotide sequence located upstream of the gene that work together with the functional gene. They can bind to the transcription factor and then play an important role. In this study, we examined the 2 kb upstream sequences of the quinoa CAMTA genes for the analysis of cis-acting elements. The results showed (Fig. 3, Table S4) that there are 10 phytohormone response elements in the promoter region: TCA-element and SARE respond to salicylic acid, ABRE responds to abscisic acid, TGA-element and AuxRR-core respond to auxin, TGACG-motif and CGTCA-motif respond to methyl jasmonate, and GARE-motif, P-box, and TATC-box respond to gibberellin. 3 elements were related to stress response: the LTR element responds to low temperature, the MBS element responds to drought, and TC-rich repeats are involved in defense and the stress response. 5 elements were related to tissue-specific expression: ARE is related to anaerobic induction, GCN4_motif is related to endosperm tissue expression, NON-box is related to specific activation of the meristem, O2-site is related to rice protein metabolism regulation, and RY-element is related to seed-specific regulation. In addition, some cis-acting elements have gene specificity. For example, O2-site only exists in *CqCAMTA01*, RY-element only exists in *CqCAMTA06*, SARE only exists in *CqCAMTA02*, and GARE-motif only exists in *CqCAMTA05*. *CqCAMTA05* (25) and *CqCAMTA07* (24) have the largest number of cis-acting elements, and *CqCAMTA02* (4) has the fewest cis-acting elements. The above analysis indicates that the 7 CAMTA genes of quinoa may be involved in the regulation of plant growth and development, phytohormonal response to stress, and other physiological processes.

**Fig. 4:**
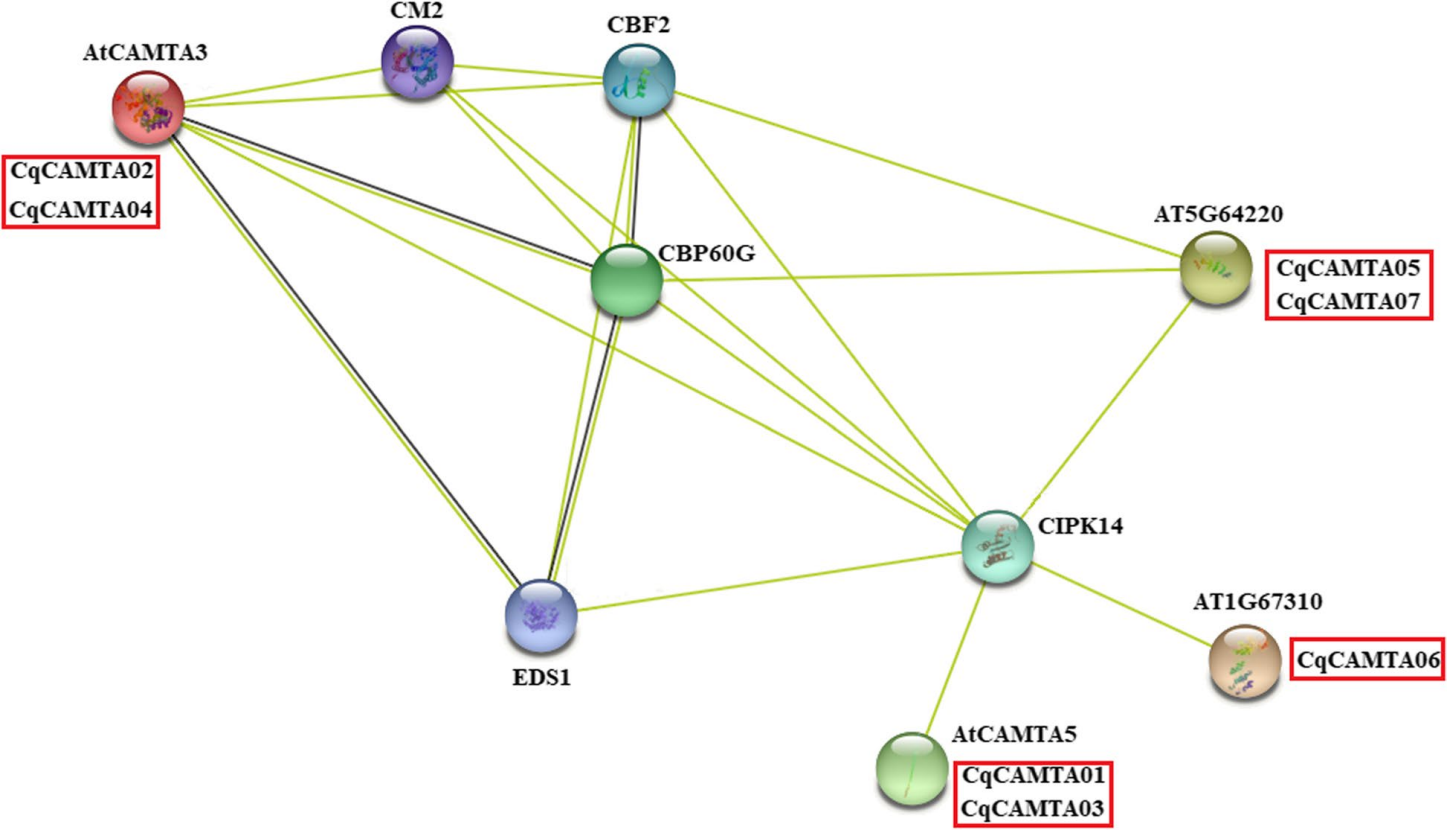
The prediction of the interaction network of CqCAMTA proteins based on the interactions of their orthologs in Arabidopsis

Interactions between proteins are integral to the activity and mechanism of transcription factors. In order to further clarify the regulatory function of CAMTA, it is necessary to analyze its interacting proteins. Using STRING 11.0 software, we constructed a quinoa CAMTA protein interaction network based on the *Arabidopsis* homologous protein interaction data to systematically analyze the mechanism of action of the 7 CqCAMTA. We found that 7 CqCAMTA proteins all appeared in the known *Arabidopsis* CAMTA protein interaction network diagram (Fig. 4). CqCAMTA02 and CqCAMTA04 are homologous to AtCAMTA3, and AtCAMTA3 mediates the cold tolerance associated with AtCAMTA1 and AtCAMTA2. It is necessary for cold-induced expression of the DREB1B/CBF1, DREB1C/CBF2, ZAT12, and GOLS3 genes. Moreover, AtCAMTA3 and AtCAMTA5 together promote the cold-induced expression of the above six genes, and thus CqCAMTA02 and CqCAMTA04 may have similar functions [49]. CqCAMTA01 and CqCAMTA03 are homologous to AtCAMTA5. AtCAMTA5 can regulate the expression of AVP1 in pollen. AVP1 regulates plant growth and development by regulating the transport of auxin [22], so CqCAMTA01 and CqCAMTA03 may also participate in plant growth and development. CqCAMTA05 and CqCAMTA07 are homologous to AT5G64220. CqCAMTA06 is homologous to AT1G67310. CBP60G seems to be the center of interaction with other genes (AtCAMTA3, CM2, CBF, EDS1, CIPK14, and T5G64220), which indicates that they may perform multiple functions as a complex. The analysis of the CqCAMTA proteins interaction network provides new research ideas for future exploration of the stress response mechanism mediated by the quinoa CAMTA transcription factors.

### qRT-PCR analysis of expression patterns of CqCAMTA under 20% PEG

**Fig. 5:**
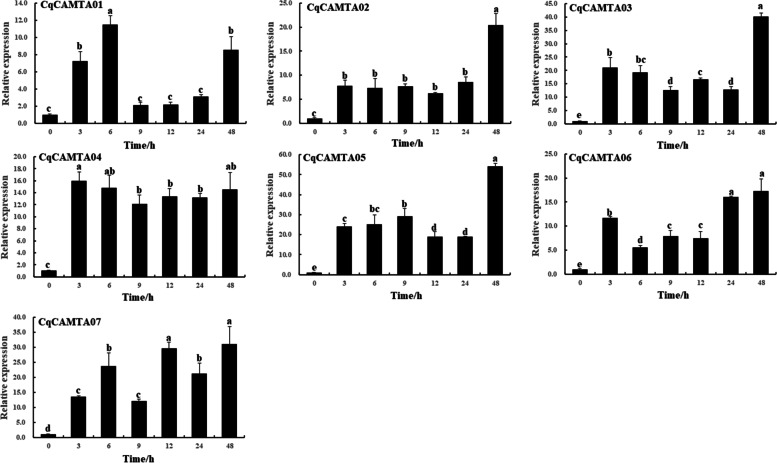
The expression levels of seven CqCAMTA genes in quinoa under 20% PEG treatments. Bars represent the mean values of three replicates ± standard deviation (SD). *CqTUB-9* is an internal reference gene

It has been reported that CAMTA genes were involved in plant response under abiotic stresses [18, 21, 23]. Therefore, the expression profiles of CqCAMTA genes under stress conditions were analyzed. In this study, qRT-PCR technology was used to detect the expression patterns of seven CqCAMTA genes under 20% PEG stress (Fig. 5, Table S2). Our results suggested that seven CqCAMTA genes responded to 20% PEG stresses. Under PEG stress, the *CqCAMTA01* gene showed no significant difference (*p* < 0.05) at multiple time points after treatment (9, 12, and 24 h) compared with 0 h (*p* < 0.05). The response of *CqCAMTA01* gene to PEG was relatively weak, while the relative expression of the other six genes (*CqCAMTA02-CqCAMTA07*) after PEG treatment was significantly up-regulated (*p < 0.05*), and the relative expression of five *CqCAMTA* genes (except *CqCAMTA01* and *CqCAMTA04*) reached the maximum, at least 15 times more than the control, after PEG treatment for 48 h. In addition, we can observe that the relative expression of most CqCAMTA genes showed a rapid increase in the first 3 h, changed little until 24 h, and rapidly increased again until 48 h. In our study, the expression levels of the seven CqCAMTA genes under 20% PEG stresses were significantly up-regulated at different time points after treatment, indicating that the members of this gene family respond to PEG stress to varying degrees.

**Fig. 6:**
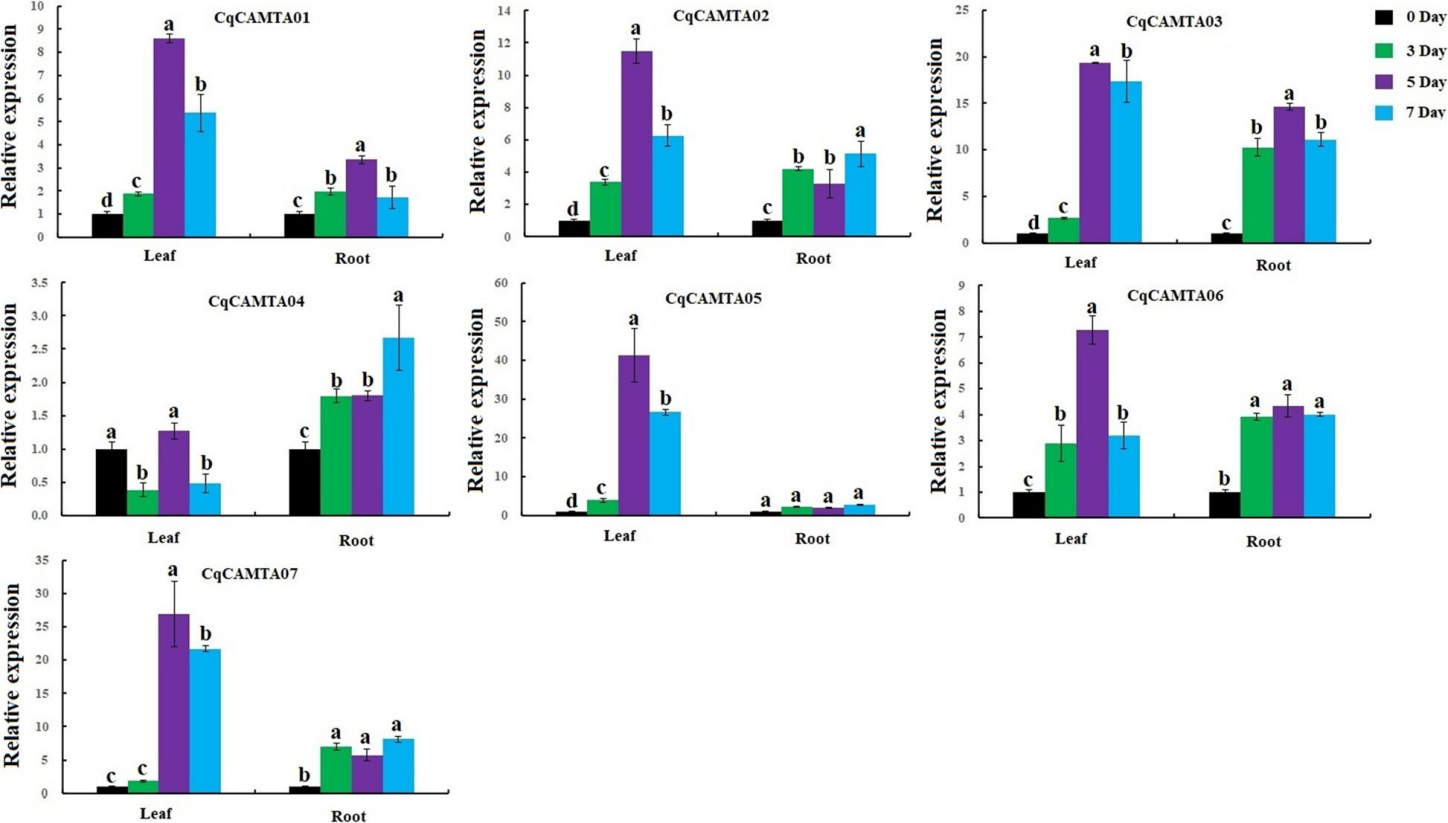
The expression levels of seven CqCAMTA genes of quinoa under drought treatments in leaf and root

In addition, we also studied the expression of *CqCAMTA* family genes in quinoa leaves and roots under drought stress (Fig. 6, Table S2). In leaves, the relative expression levels of six *CqCAMTA* genes (except *CqCAMTA04*) showing a similar expression pattern (increasing first and then decreasing) with the duration of drought days, and expression levels increased relatively slowly in the first 3 d and then increased relatively sharply until day 5, when they reached their maximum, especially the *CqCAMTA03*, *CqCAMTA05* and *CqCAMTA07* genes, and the expression levels of all 6 genes showed a downward trend within 5 to 7 d. In roots, our results illustrated that the expression of the *CqCAMTA05* gene was not significantly different before and after drought stress, indicating that this gene did not respond to drought stress in roots. The remaining 6 CqCAMTA genes had significant differences in roots after drought treatment and the control. Among them, *CqCAMTA01*, *CqCAMTA02*, *CqCAMTA04*, *CqCAMTA06,* and *CqCAMTA07* genes were all up-regulated in the drought-treated roots, but the degree of up-regulation was low. The relative expression of *CqCAMTA03* gene at various time points (3, 5, and 7 d) after drought treatment was significantly different from that of the control, and was at least 10 times that of the control.

### Subcellular localization and characterization of transcription activity of *CqCAMTA03*

**Fig. 7:**
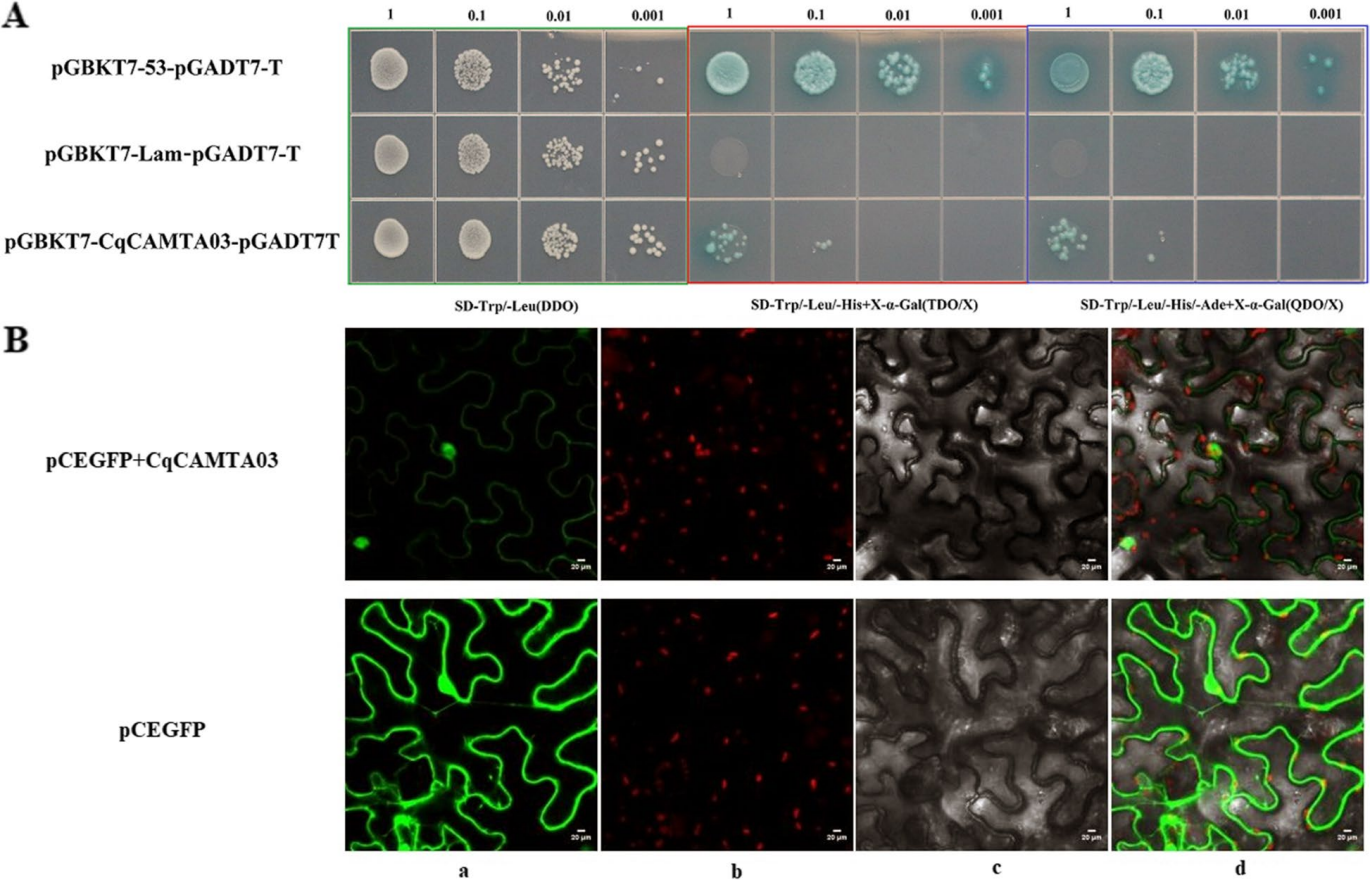
Autonomous transcriptional activation testing of pGBKT7-CqCAMTA03 and Subcellular localization of pCEGFP-CqCAMTA03. **A** Autonomous transcriptional activation testing. **B** Subcellular localization of CqCAMTA03. **a**-**d** separately represent the GFP green fluorescent protein, chloroplast autofluorescence, bright field, and the overlay of three channels

The yeast strains successfully transformed with the pGBKT7-CqCAMTA03 bait expression vector were diluted and spread on three defective solid media SD-Trp/−Leu, SD/−Trp/Xa-Gal, SD/−Trp/Xa-Gal. They were cultured for 2–3 d, and growth was observed. The results of the self-activation test of the bait vector showed (Fig. 7A) that the yeast containing the positive control vector could grow on all three media; the negative control only grew on SD-Trp/−Leu media, containing pGBKT7. Yeasts containing pGBKT7-CqCAMTA03 could grow normally on all three media. The experimental results showed that the pGBKT7-CqCAMTA03 gene had transcriptional auto-activation.

The subcellular localization prediction results showed that the CqCAMTA03 protein was localized in the nucleus (Fig. 7B). To further verify the localization of CqCAMTA03 in cells, we carried out the subcellular localization test in tobacco. The *Agrobacterium* successfully transformed with 35S-CqCAMTA03-YFP and 35S-YFP vectors were injected into tobacco, and the distribution of fluorescent proteins was examined under a confocal microscope. The prediction results illustrated that CqCAMTA03 protein was localized in the nucleus.

### Response of the overexpressed lines and the wild type under drought stress conditions

**Fig. 8:**
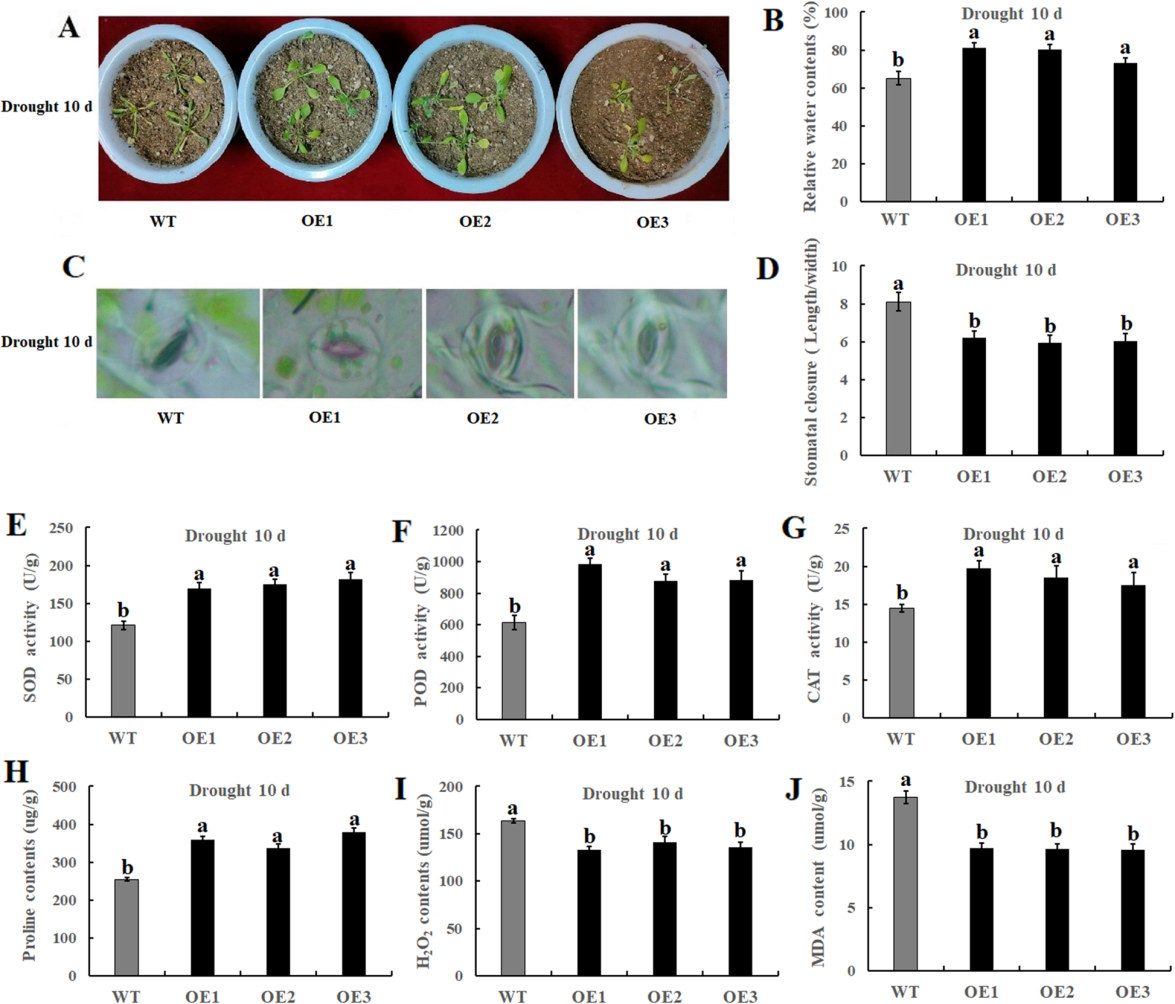
Determination of physiological traits. **A** Transgenic *Arabidopsis* lines and wild type under drought conditions; **B** Quantitative determination of relative water content (RLWC); **C** Images of stoma in the target gene-overexpressed and WT plants under drought stress; (**D**) Comparative stomatal aperture measurements. **E**-**G** SOD, POD and CAT activity; (**H**-**J**) Proline, H_2_O_2_ and MDA content

In order to further study the function of the *CqCAMTA03* gene, we used transgenic technology to overexpress *CqCAMTA03* gene in *Arabidopsis thaliana*, and further studied the function of this gene in drought stress. The results indicated that the overexpressed *Arabidopsis* lines were more tolerant to water-deficient conditions, as demonstrated with several physiological indicators. For example, the relative water content of the overexpressed lines after drought stress was significantly higher than that of the WT (Fig. 8A-B); in addition, the *CqCAMTA03* overexpressed plants reduced the degree of stomatal opening in response to drought stress (Fig. 8C-D). We detected the levels of three antioxidant enzymes POD, SOD, and CAT (Fig. 8E-G); the results suggested that the activities of the three antioxidant enzymes in transgenic *Arabidopsis* leaves under drought stress were significantly higher than those in the WT. Proline content in the overexpressed plants was also significantly higher than that in the WT (Fig. 8H). Malondialdehyde (MDA) levels are a key indicator of lipid degradation due to oxidation from excess release and accumulation of reactive oxygen species. In this study, MDA and H_2_O_2_ contents in *Arabidopsis* lines overexpressing the *CqCAMTA03* gene were significantly lower than those in wild *Arabidopsis* plants after drought stress (Fig. 8I-J), indicating that the *Arabidopsis* lines overexpressing *CqCAMTA03* gene had high tolerance to oxidative stress, thereby minimizing oxidative damage.

## Discussion

Drought, low temperatures, saline-alkali conditions, and various hormone stresses can cause specific changes in intracellular Ca^2+^ concentration. CaMs/CMLs are crucial Ca^2+^ sensors that can decode the information carried in these Ca^2+^ signals and convert specific Ca^2+^ signals to an appropriate downstream effector [8]. CAMTA, WRKY, and MYB in plants can interact with CaMs/CMLs and play key roles in the response of plants to various stress [50]. The CAMTA gene has been identified in a variety of plants and is involved in a variety of stress responses as well as growth [50]. However, the gene family had not yet been reported in quinoa. Therefore, this study used the quinoa genome as a reference, and a total of seven members of the CqCAMTA gene family were identified in quinoa. The sequence and structural pattern of the CqCAMTA proteins are highly similar, indicating that these CqCAMTA genes may be derived from an ancestral sequence. The analysis of basic characteristics showed that the amino acid number of CqCAMTA is 931aa − 1000 aa, which is similar to those found in *Arabidopsis* [17], rice [14], tomato [18], corn [19], flax [20] and banana [21]. The six CqCAMTA proteins have an isoelectric point less than 7 (except CqCAMTA01), making them acidic proteins. However, in the report of 15 wheat TaCAMTA genes by Yang [51], six of the TaCAMTA genes are composed of basic amino acids, accounting for 40% of the total. Pant [52] studied the three species of *Gossypium* (which harbor 6, 7, and 9 GhCAMTA genes) and found 2, 4, and 2 genes composed of basic amino acids, with the proportions of 33, 57, and 22%. These findings indicate that the proportions of basic and acidic amino acids in the CAMTA gene family differ across species. The subcellular location showed that the seven CqCAMTA genes were found in the nucleus, and similar results were found in wheat [51], indicating that they play a role in regulating the expression of other genes. Based on the CqCAMTA protein sequence, a complete phylogenetic tree of quinoa, *Arabidopsis*, pepper, spinach, and grape was established to research the relationship of CAMTA genes among these five species. Based on phylogenetic analysis, we found that there are 3 pairs of paralogous genes in quinoa CAMTA, indicating that these 3 pairs of CAMTA genes have similar functions. However, C*qCAMTA06* forms an independent branch, indicating that it was isolated during the evolution of quinoa. In addition, proteins with similar conserved motifs and conserved domains in CqCAMTAs members are clustered in the same group. For example, *CqCAMTA01*/*CqCAMTA03* clusters in Group A, while *CqCAMTA02*/*CqCAMTA04*, *CqCAMTA05*/*CqCAMTA07* clusters in Group B. This is similar to the phylogenetic evolutionary relationship observed in tobacco and cotton [11, 52]. It is speculated that genes in the same subtribe have similar structure, function, and evolution characteristics.

The study of exons and introns helps to further understand the differences in gene structure and function among gene family members [51]. In our study, CqCAMTA genes located in the same branch have similar numbers of exons and arrangement patterns, and the number of exons of the CqCAMTA gene family is between 12 and 13. Similar CAMTA genes have been observed in various plants [19, 51], and this fixed number of introns-exons is a conserved feature of CAMTAs, indicating that CAMTA genes are conserved across species. Additionally, the study of protein structure is necessary to explain mode of action, so we examined the basic motifs of quinoa CAMTAs proteins. The proteins encoded by plant CAMTAs have four functional domains: CG-1, ANK, IPT/TIG, and IQ motif protein-binding [20]. The main basic domains, such as CG 1, ANK, IPT/TIG, and IQ, are found in the *CqCAMTA* genes, which are highly conserved throughout the species [11, 51, 52]. The conserved domains of CqCAMTA proteins also showed similar results in the same subgroup. Analysis of cis-regulatory elements showed that CqCAMTA proteins are transcriptionally regulated under abiotic stresses including anaerobic, drought, and low temperature conditions, and participate in the signal transduction regulation of hormones such as auxin, gibberellin, and salicylic acid. Some genes have response sites in callus response elements, endosperm tissue expression, and seed-specific regulatory element expression levels, but not all of them can effectively bind and affect expression. If they do affect expression, regulation can be both positive and negative. MBS (drought-induced response) elements exist in *CqCAMTA01* and *CqCAMTA03*, and MBS has been used to study the molecular mechanism of drought.

Many life stages and processes may be affected. CAMTA/SR mediates plant growth, *AtCAMTA1* and *AtCAMTA5* can regulate the expression of *AVP1* in pollen, and *AtCAMTA3/SR1* participates in plant growth and development through signal pathways mediated by auxin and BR [22]. In addition, Ca^2+^ is related to fruit ripening. Yang [18] found that seven CAMTA/SRs of tomato are differentially expressed during fruit development and maturation. CAMTA/SR may be used as a node for developmental signals, calcium signals, and ethylene signals in tomato fruits. It is critical in development and maturity. Yang [18] verified through ChIP experiments that *AtCAMTA3/SR1* can bind to the EIN3 (ethylene insensitive 3) promoter region and directly participate in the aging process caused by ethylene. The expression analysis in that study showed that most CqCAMTAs can be expressed in a variety of tissues (root and leaf), indicating that CAMTA might mediate the growth of quinoa and other plants, a similar finding was made in citrus [18].

CAMTA genes can help plants cope with various environmental and hormonal stresses [23–28]. ABRE (ACGTGT) is an abscisic acid response element identified by Doron [27] in the promoter of HKT1; it serves as a key factor in response to NaCl, Drought and ABA stress. The ABRE element can be the target of various types of TF, which shows that the ABRE element is essential in increasing the expression of this gene under NaCl, Drought and ABA stress. In this study, we also found that the *CqCAMTA07* gene strongly responds to20% PEG stress in leaves. Further, the promoter region of this gene contains 7 ABA response elements (ABREs). It is therefore speculated that this gene may regulate the response to drought stress during seedling growth through the ABA signaling pathway. Mutants of the *AtCAMTA01* gene in *Arabidopsis* showed a high degree of drought sensitivity, indicating that this gene acts in a positive regulatory role in drought response. *AtCAMTA1* does so by modulating the expression of transcription factors and the ABA response. In addition, *CAMTA1* also regulates some genes related to stress, including *RD26*, *ERD7*, *RAB18*, and *LTPs*. *CAMTA1* may alleviate the adverse effects of drought by regulating AP2-EREBP gene expression and the ABA response [24]. *MuCAMTA1* is up-regulated 40 times after drought stress, which may make it an ideal gene to improve banana drought resistance [21]. Overexpression of *GmCAMTA12* in soybeans improves the drought resistance of hairy roots [26]. In this study, CqCAMTA family members responded to drought stress to varying degrees. The interaction network diagram showed that CqCAMTA homologous genes in *Arabidopsis* all interact with *AtCIPK14*. Studies have shown that [53] *AtCIPK14* can positively regulate responses to drought, low temperature, salt, alkali and other abiotic stresses in pigeon pea, while *CcCIPK14*-*CcCBL1* positively regulates drought tolerance by enhancing the biosynthesis of flavonoids. Therefore, the mechanism of CqCAMTA response to drought may be that this family of genes mediates the CIPK signaling pathway. In addition, the expression of *CqCAMTA03* gene in roots and leaves increased significantly at various time points after drought stress, indicating that the gene responds strongly to drought stress. This could be due to its promoter region containing MBS elements (which are involved in the response to drought), and/or because the promoter region contains ABRE and TCA-element hormone response elements (which are involved in the response to ABA and SA). Studies have shown that *AtCAMTA1* can alleviate drought pressure by regulating the expression of TFs and ABA response, and *AtCAMTA3* is sensitive to drought stress, because it is related to the excessive accumulation of SA [22].

Drought stress primarily affects plant photosynthetic efficiency through stomatal closure. However, some degree of stomatal opening is beneficial to plant growth under drought stress [54]. Under drought conditions, the apple cultivar “Qin Guan” with high WUE had larger stomatal aperture than did “Mi Su” with low WUE. In this study, we found that under drought stress, the stomatal aperture of the CqCAMTA03-OE strain was larger than that of the WT strain. Antioxidant enzymes, such as CAT, POD, and SOD, play an important role in the excessive accumulation of reactive oxygen species (ROS). ROS can damage the plant cell membrane system [55]. In this study, the CqCAMTA03-OE strain had higher CAT, POD, and SOD activities, and lower H_2_O_2_ content, than did the WT strain under drought stress. These findings indicated that overexpression of CqCAMTA03 enhanced the antioxidant system. In addition, osmotic regulation is an important drought adaptation strategy to support plant growth and development. Under drought stress, plants accumulate various compatible solutes (e.g. proline) for osmotic adjustment [56]. In this study, the CqCAMTA03-OE strain accumulated more proline than did the WT strain, resulting in enhanced osmotic regulation in response to drought stress. In conclusion, these results suggested that CqCAMTA03 is a positive regulator of drought tolerance in quinoa, and provided a framework for further understanding this important crop.

## Supplementary Information

**Additional file 1: Table S1.** GFF annotation information of seven CqCAMTA genes.

**Additional file 2: Table S2.** The primer designed for qRT-PCR.

**Additional file 3: Table S3.** The 27 CAMTA gene-coding protein sequence information in this study.

**Additional file 4: Fig. S1.** The conserved domain of seven CqCAMTAs.

**Additional file 5: Fig. S2**. The distribution of seven CqCAMTAs on the chromosome.

**Additional file 6: Table S4.** cis-acting elements in the promoter region of CqCAMTA genes.

## Data Availability

The reference genome assembly used for data analysis was obtained from National Center for Biotechnology Information (NCBI). The datasets analysed during this study are included in this published article and its supplementary information files.

## Abbreviations

*ABA*: Abscisic acid
*qRT-PCR*: Quantitative RT-PCR
*UTR*: Untranslated region
