# Supplementary figures and images for: Characterization of the CqCAMTA gene family reveals the role of CqCAMTA03 in drought tolerance
Source: BMC Plant Biol. 2022 Sep 7;22:428. doi: 10.1186/s12870-022-03817-0 (PMC9450354; doi:10.1186/s12870-022-03817-0)

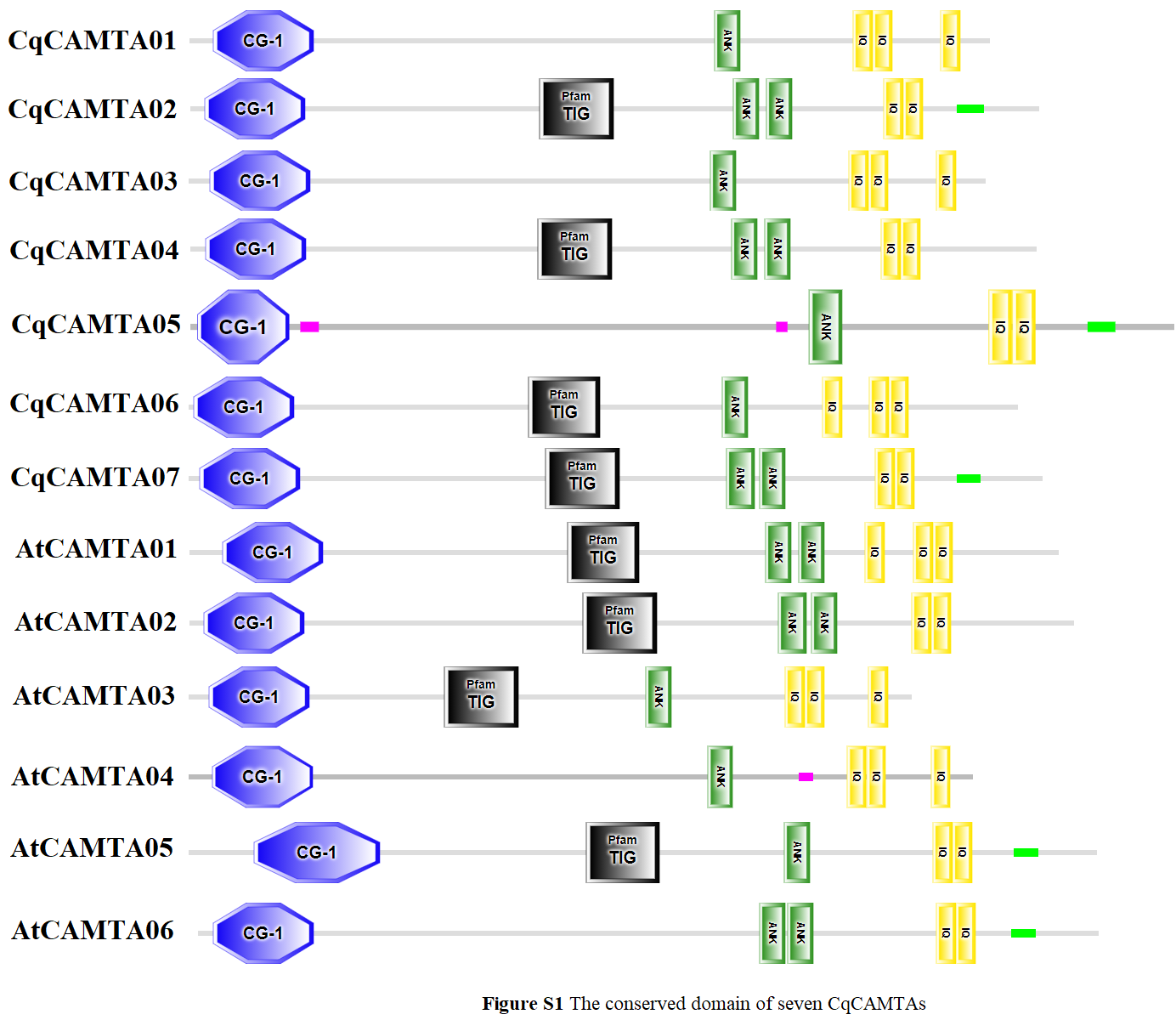

Supplement: Supplementary file 4 — Additional file 4: Fig. S1. The conserved domain of seven CqCAMTAs. [file 12870_2022_3817_MOESM4_ESM.tif]

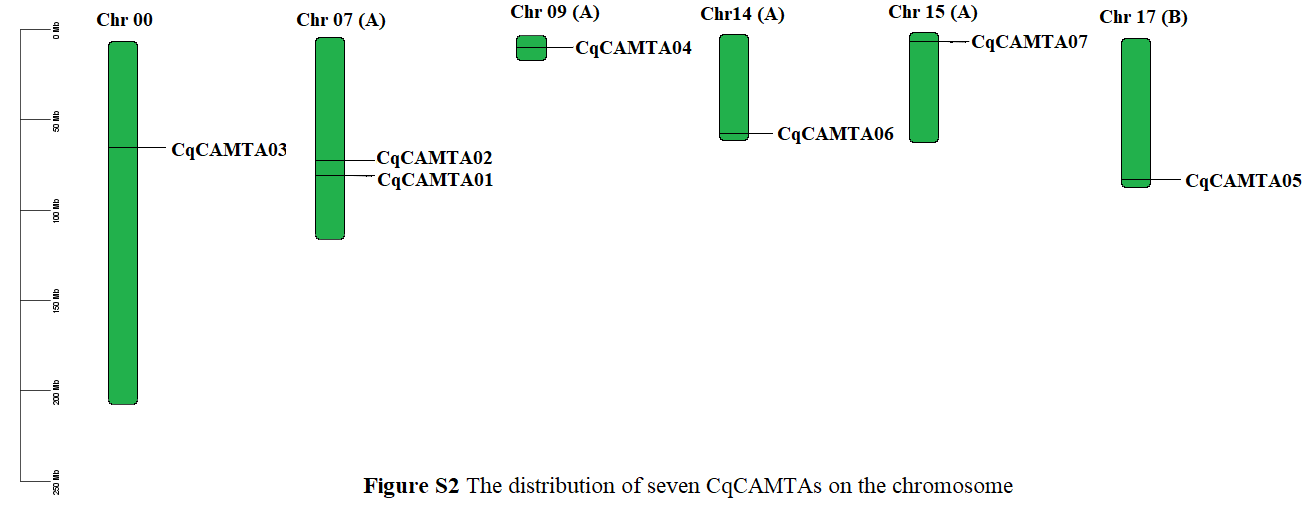

Supplement: Supplementary file 5 — Additional file 5: Fig. S2. The distribution of seven CqCAMTAs on the chromosome. [file 12870_2022_3817_MOESM5_ESM.tif]
